# The Fiber Optic Reel System: A Compact Deployment Solution for Tethered Live-Telemetry Deep-Sea Robots and Sensors

**DOI:** 10.3390/s21072526

**Published:** 2021-04-04

**Authors:** Brennan T. Phillips, Nicholas Chaloux, Russell Shomberg, Adriana Muñoz-Soto, Jim Owens

**Affiliations:** 1 Department of Ocean Engineering, University of Rhode Island, Narragansett, RI 02882, USA; nchaloux@uri.edu (N.C.); rshomberg@uri.edu (R.S.); 2 Graduate School of Oceanography, University of Rhode Island, Narragansett, RI 02882, USA; 3 Department of Mechanical Engineering, University of Puerto Rico Mayaguez, Mayagüez, PR 00681, USA; adriana.munoz2@upr.edu; 4 National Oceanic and Atmospheric Administration, EPP/MSI Undergraduate Scholarship Program, Silver Spring, MD 20910, USA; 5 Nautilus Defense LLC, Pawtucket, RI 02860, USA; jim@nautilusdefense.com

**Keywords:** fiber optics, deep-sea robotics, distributed sensing, remotely operated vehicles

## Abstract

Tethered deep-sea robots and instrument platforms, such as Remotely Operated Vehicles (ROVs) and vertical-profiling or towed instrument arrays, commonly rely on fiber optics for real-time data transmission. Fiber optic tethers used for these applications are either heavily reinforced load-bearing cables used to support lifting and pulling, or bare optical fibers used in non-load bearing applications. Load-bearing tethers directly scale operations for deep-sea robots as the cable diameter, mass, and length typically require heavy winches and large surface support vessels to operate, and also guide the design of the deep-sea robot itself. In an effort to dramatically reduce the physical scale and operational overhead of tethered live-telemetry deep-sea robots and sensors, we have developed the Fiber Optic Reel System (FOReelS). FOReelS utilizes a customized electric fishing reel outfitted with a proprietary hollow-core braided fiber optic fishing line and mechanical termination assembly (FOFL), which offers an extremely small diameter (750 μm) load-bearing (90 lb/400 N breaking strength) tether to support live high-bandwidth data transmission as well as fiber optic sensing applications. The system incorporates a novel epoxy potted data payload system (DPS) that includes high-definition video, integrated lighting, rechargeable battery power, and gigabit ethernet fiber optic telemetry. In this paper we present the complete FOReelS design and field demonstrations to depths exceeding 780 m using small coastal support vessels of opportunity. FOReelS is likely the smallest form factor live-telemetry deep-sea exploration tool currently in existence, with a broad range of future applications envisioned for oceanographic sensing and communication.

## 1. Introduction

Human access to the deep-sea is facilitated through a spectrum of platforms such as untethered human-occupied vehicles (HOVs), unmanned underwater vehicles (UUVs) and a wide variety of cabled/tethered systems such as ROVs [1]. Tethered systems employing fiber optics offer a tremendous advantage due to their ability to transmit large quantities of data, including live video, over great distances with extremely low latency [2]. Modern deep-rated ROVs, towsleds, and sonar systems (>1000 m cable length) almost exclusively employ fiber optics for data transmission, typically via steel-armored cables that include power transmission over copper conductors. The size of these cable spools often dwarf the subsea vehicles themselves and require large oceanographic-class winches installed on appropriately sized support vessels for operation. A notable exception is the recent development of ‘hybrid’ ROVs which instead rely on battery-powered vehicles and bare optical fiber that is free-spooled at almost zero tension throughout the course of a vehicle deployment [3,4,5,6,7]. ‘Hybrid’ ROVs, which can operate as both tethered ROVs and untethered UUVs, have demonstrated their efficacy at the deepest depths of the ocean [8,9]. In these systems a major length of the tether is bare optical fiber that is extremely fragile, non-load bearing, and cannot be reused for multiple deployments; the robot is retrieved at the surface as a free-swimming untethered autonomous system.

The scale of an ROV in terms of physical size, mass, and power requirements is a major driver in the overall operating scheme of deep-sea tethered systems. Modern work-class ROVs can weigh several tons and require industrial-scale lifting equipment for launch and recovery. The aforementioned ‘hybrid’ ROVs, while less reliant on a large surface winch, still must include a large battery bank for sustained power and often require the use of a relatively large support vessel to launch and recover the vehicle itself, with some exceptions [10]. The major electronic components of contemporary ROVs are almost exclusively packaged in pressure housings, further adding to their size and weight. These large and sophisticated systems are also known to cause avoidance effects amongst deep-sea mobile faunas, particularly in the midwater environment. Such avoidance effects are most likely due to artificial lighting schemes which can be addressed with wavelength-specific lights [10,11], but there is some evidence of avoidance associated solely to the presence of a large physical shape [12]. Recent advances in pressure-tolerant electronics as well as 3D-printed, epoxy-filled assemblies [13] reduce the reliance on pressure housings and are leading the way towards ultra-compact, self-powered subsea systems that can operate in depths exceeding 1000 m. Such compact designs are well-suited to a low-profile tether design but cannot support specialized Neried-class fiber optic deployment architecture.

In this paper we present an innovative approach to tethered deep-sea vehicles via a “fiber optic reel system” (FOReelS). FOReelS relies on a novel load-bearing 750 μm diameter singlemode fiber optic cable based on hollow-core braid fishing line, hereafter referred to as ‘fiber optic fishing line’ (FOFL), which can be used to conduct repeated deployments and recoveries using the same tether. FOFL also has design properties that are uniquely suited for distributed acoustic and temperature sensing (DAS/DTS). A 3D-printed, epoxy-filled mechanical termination isolates tensile loading on the FOFL from the optical fiber and has a demonstrated breaking strength of 90 lbs (400 N). The termination is connected to a custom 3D-printed, epoxy-potted data payload system (DPS) based on the DEEPi camera system [13] and uses a rechargeable battery and an ethernet-to-fiber converter to achieve bidirectional gigabit telemetry. The FOReelS system is described in detail, along with field results demonstrating live HD video transmission from up to 780 m deep using small support vessels of opportunity. Future steps include adding additional sensors, navigational equipment, and small thrusters to the subsea payload to achieve full mobility as a deep-sea ROV. To our knowledge FOReelS is the smallest, lightest, and least expensive deep-sea live telemetry system created to date and represents a translational leap forward in the design of tethered deep-sea robotic systems.

## 2. Materials and Methods

The FOReelS system was developed using a combination of commercial off-the-shelf components, custom-manufactured parts, and 3D-printed elements. The fiber optic fishing line (FOFL) consists of an outer sheath comprised of eight 400 denier UHMWPE yarns biaxially braided in a diamond configuration and an inner core comprised of a single Corning SMF-28e+ bare single mode fiber optic. The FOFL was constructed using a custom maypole braiding machine designed to ensure that the minimum bend radius of the fiber optic core was not exceeded, while also limiting the tension on the fiber optic core throughout manufacturing. Tensile testing was performed using a Shimadzu AGS-X 10kN electromechanical test frame, with reference to ASTM Standard F3410-19 to guide rate and loading procedures.

The mechanical termination housing was produced using a Formlabs Form 2 and Form 3 stereolithography 3D printers using Standard Clear resin. The epoxy potting compound used inside the termination was Crystal Clear 202 (Reynolds Advanced Materials, Brighton, MA, USA). The furcation tube was type 304 stainless-steel needle tubing (McMaster-Carr, Elmhurst, IL, USA). The DPS was comprised of several major electronic components mounted inside a 3D-printed mold and potted in epoxy. The control computer was a Raspberry Pi Model 4 B with 2 GB RAM and a 256 GB microSD card. The battery was a 4400 mAh 3.7 V pack (Adafruit, New York, NY, USA). The fiber optic media converter was a 10 Gtek 1.25 G outfitted with a bidirectional 1510 nm SFP transceiver module. The imaging sensor was a Raspberry Pi Camera Module V2, and the light was a modified 450 lumen Actik Core LED headlamp (Petzl America, West Valley City, UT, USA). The chassis and housing was produced using a Formlabs Form 2/Form 3 stereolithography 3D printer using Standard Clear resin. The epoxy potting compound used inside the termination was Crystal Clear 200 and 202 (Reynolds Advanced Materials, Brighton, MA, USA). Battery charging was achieved using a MacArtney/Subconn MCBH4F bulkhead connector. Depth, temperature, attitude and compass data were recorded using a Star-Oddi DST Compass Magnetic unit. A Lindgren-Pitman S1200 electric fishing reel was heavily modified as described in the Results section. The slipring used on the reel was a singlemode/single-pass RPCA-155-28A-STX model produced by Princetel, Inc., Hamilton Township, NJ, USA. 

## 3. Results

### 3.1. FOFL and Mechanical Termination

**Figure 1:**
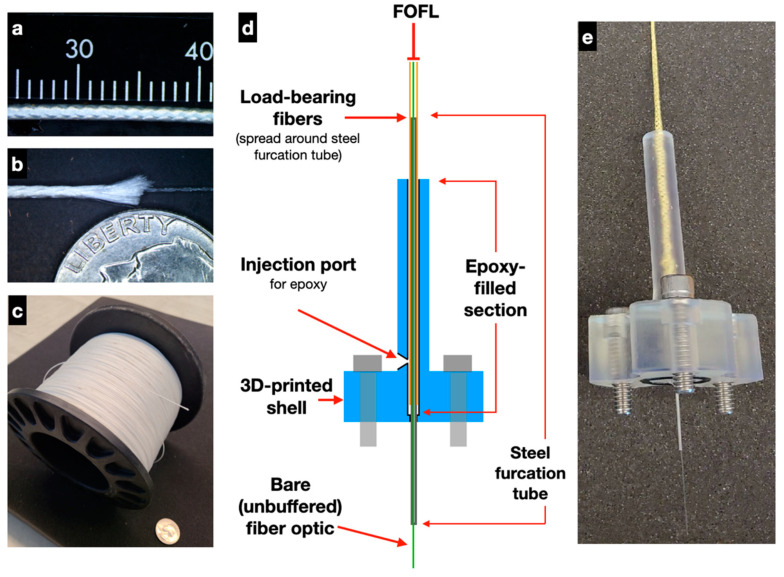
(**a**) Fiber optic fishing line (FOFL) comprised of UHMWPE load-bearing fibers braided around singlemode fiber optic. (**b**) View of FOFL with braided fibers cut at an end, exposing the fiber optic core, with a quarter shown for scale. (**c**) A spool of approximately 1100 m of FOFL wound on a electric fishing reel drum, with a 100 mm core diameter and 100 mm flange width. (**d**) Cross-sectional diagram of FOFL mechanical termination showing major components and epoxy-filled section inside 3D-printed housing. (**e**) FOFL mechanical termination, shown with an early prototype FOFL using Kevlar load-bearing fibers for the outer braid.

The design of FOFL is based on hollow-core braid fishing line that is commercially available (e.g. Tuf-Line XP, Mustad & Sons, Miami, FL, USA) typically constructed using aramid/Kevlar or UHMWPE fibers. An early prototype version of FOFL based on Tuf-Line XP (250 lb test) was produced by Mustad’s subsidiary company Western Filament, Inc.; in parallel, custom designs were pursued by Nautilus Defense LLC. The resulting design of FOFL is an 8-strand UHMWPE construction that is braided around an unbuffered singlemode fiber (Corning SMF-28e+) (Figure 1a). The FOFL’s braided sheath (750 μm outer diameter) has a minimum inner diameter when under tension that is greater than the outer diameter of the incorporated fiber optic core. This reduces mechanical coupling between the fiber optic core and braided sheath due to compressive forces on the core from the sheath. The minimum inner diameter of the braid is determined by the size and number of the yarns comprising it and its geometry when in a jammed state. The jammed state of the braid is the condition where the angle at which the braid’s yarns intersect cannot decrease any further when the braid undergoes axial compression or tension. Axial tension is the modality of concern for the FOReelS system. Given the extreme flexibility afforded by the hollow-core braid, it is assumed that the minimum bend radius of this optical cable is 16 mm, the same as bare optical fiber itself.

The FOFL mechanical termination is a custom design that employs a 3D-printed housing, stainless-steel furcation tube, and rigid fast-cure epoxy to isolate tensile loading from the fiber optic core (Figure 1b). To assemble, the FOFL outer braid is initially trimmed ~20 cm back from the bitter end to expose a usable length of fiber optic. A stainless-steel furcation tube (1 mm OD, 0.2 mm wall thickness, 10 cm length) is then inserted over the optical fiber and along the inside of the braid. This assembly is then inserted into the 3D-printed termination piece so that the stainless-steel tube passes completely through, and a small amount of hot glue is used to seal the lower-outside portion of the tube against the bottom of the termination piece. Finally, uncured epoxy is injected into the termination piece, filled to the top, and the injection port sealed with electrical tape to prevent the epoxy from flowing back out. Once the epoxy has cured, the FOFL is fully bonded to the termination piece while the fiber optic is allowed to move freely inside of the stainless-steel tube. Several layers of heat shrink are typically used as a bending strain relief at the top of the termination piece.

Tensile testing of the FOFL mechanical termination was performed using two double-ended specimens, with the ends of each FOFL specimen prepared in the same fashion as all field-tested prototypes (Video S1). These two specimens failed at 87 and 91 lbs (387, 404 N), with failure occurring at the entry/exit point into the stainless-steel furcation tube. This behavior is expected, as this point in the termination is where the braid sees full tensile loading and the individual fibers are in direct contact with the metal tube edge. Other tensile tests performed on five single-ended FOFL mechanical terminations failed in the 40–75 lb (178–335 N) range, with all failures occurring at the grip point for the FOFL line. Given these results, we conclude that the mechanical termination itself is stronger than the FOFL.

### 3.2. Data Payload System (DPS)

**Figure 2:**
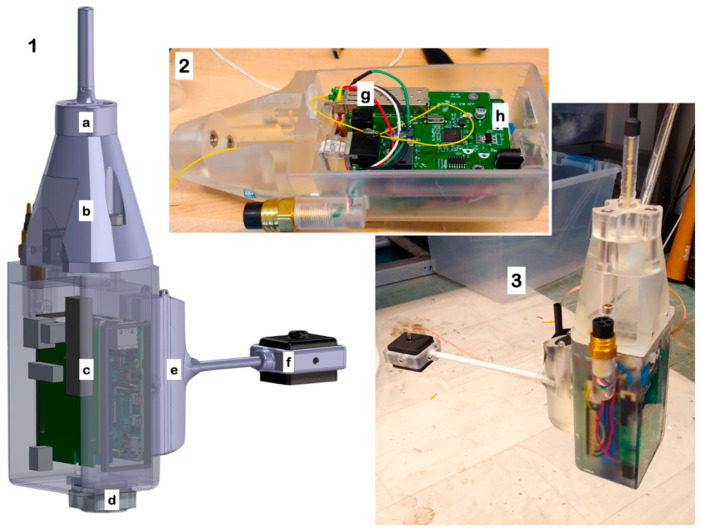
Data payload system (DPS) used with the FOReelS system. (**1**) CAD model of DPS with (**a**) FOFL mechanical termination; (**b**) water-flooded fiber optic junction box and removable cover; (**c**) potted electronics module with microcontroller, rechargeable Li-ion battery, and fiber optic to ethernet converter; (**d**) DEEPi camera head; (**e**) modular fin with removeable depth/temperature data logger and (**f**) potted LED lamp. (**2**) Partially assembled DPS prepared for epoxy potting showing (**g**) modified SFP laser module and (**h**) stacked electronics assembly on 3D-printed internal chassis. (**3**) Completed DPS prototype used in this study.

The DPS used to demonstrate FOReelS is based on previous development efforts for DEEPi, a low-cost 3D-printed, epoxy-filled camera/computer system using Raspberry Pi components. In the FOReelS version, a 4400 mAh LiIon battery (two 18,650 cells) and an ethernet-to-fiber converter is included along with a Raspberry Pi 4 B+ computer and Raspberry Pi Camera V2 (Figure 2). Livestreaming/remote connectivity is achieved using a dynamic web server hosted on the Raspberry Pi. The Raspberry Pi runs a webserver using the flask module from Python. This webserver serves a single dynamic page consisting of a ‘mjpeg’ file updated from the latest camera frame and buttons which send a limited number of preset commands to the Raspberry Pi. The frame rate is not set. Instead the mjpeg updates as quickly as the connection will allow. All code used is available as an open-source repositories (https://github.com/uril-group/DEEPi-OS and https://github.com/rshom/DEEPi-BRUV, accessed on 10 March 2021). The entire electronics assembly is mounted using a 3D-printed scaffold, which is then installed inside an outer 3D-printed mold. A standard 4-pin wet-mate underwater connector is used as a charge plug, as well as a ‘power on’ switch when two of the pins are shorted using a custom connector. The fiber optic SFP transmitter module cannot be potted directly without modification. Prior to epoxy potting, the laser module inside the SFP was coated on the exterior using hot glue. The LC optical connector was also sealed into the laser module by applying a small coat of cyanoacrylate (Loctite 416) to the ceramic ferrule. These steps seal the optical components during the potting process, and creates a small air void deep inside the epoxy.

Lighting for the DPS is achieved using a potted LED assembly that uses an off-the-shelf sporting equipment headlamp (450 lumens) for the internal electronics. The momentary switch used to select brightness levels is controlled directly through one of the Raspberry Pi’s GPIO pins. Power for the LED assembly is shared with the main battery bus, and a voltage regulator is potted in-line to maintain consistent power to the lighting array. With lighting set at maximum brightness, it is possible for the DPS to broadcast and record a video stream for over 1hr on a single charge. Recharging of the system is achieved through the wet-mateable connector using a laboratory power supply and external Li-ion charging circuit.

### 3.3. FOReelS System Description

**Figure 3:**
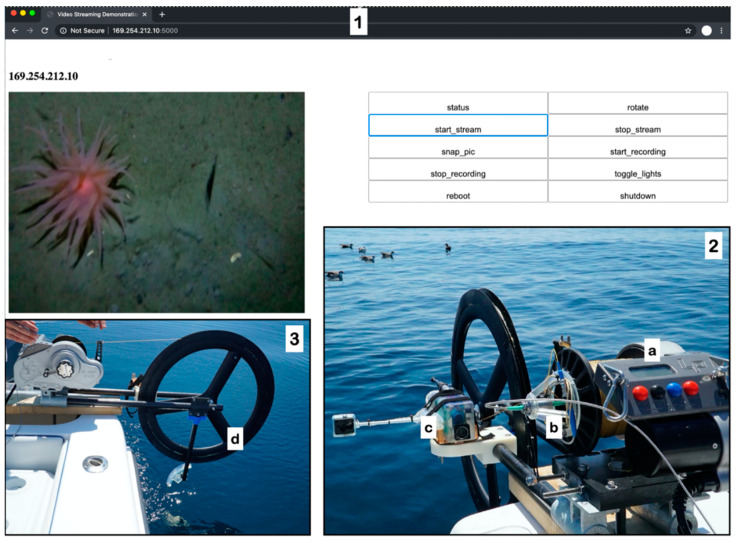
Deepwater field trials of the FOReelS system. (**1**) Screenshot of live video stream from approximately 350 m as the FOReelS is used to explore Atlantis Canyon in August 2020, including control GUI running on a Google Chrome browser window. (**2**) FOReelS system mounted on the transom of a 31′ center console fishing vessel, used for the Atlantis Canyon deepwater field trials; (**a**) commercial off-the-shelf electric fishing reel; (**b**) fiber optic slip-ring and custom 3D-printed mount; (**c**) DPS, prepared for deployment. (**3**) Side view of FOReelS mid-deployment showing (**d**) carbon fiber wheel used to increase the bending radius of the overboarding sheave.

A high-capacity electric fishing reel (Lindgren-Pitman, S1200 model) was modified to accommodate the FOFL, large-diameter overboarding sheave, and DPS (Figure 3). FOFL was wound directly onto the reel drum and a drilled hole on the inside allowed for the FOFL to pass through to the center of the drum. At least 1500 m of FOFL can fit onto a standard drum for the fishing reel. A compact singlemode fiber optic slipring was mounted on the drum using a custom 3D-printed adapter. In place of a fishing rod, a custom carbon-fiber boom outfitted with a carbon-fiber bicycle wheel was used to increase the bending radius of the FOFL under tension; laboratory tests indicated that the reel drum alone is too small diameter, causing unacceptably high attenuation levels of the optical signal. A single 12V lead-acid car battery was used to power the reel, which holds enough capacity for multiple full-cast deployments on a single charge. Topside, a fiber-to-ethernet media converter was put directly in-line with a laptop computer to communicate with the payload for livestream video and lighting control throughout the cast.

### 3.4. Field Demonstrations

To date, a total of four field trials have been conducted with the FOReelS system. Early deployments took place in Narragansett Bay, RI USA, using a 16.5′ Boston Whaler center console small boat. The first deployment to approximately 50 m depth took place off Castle Hill Lighthouse on 3 July 2020 (41.476746° N, −71.347733° W) using an early prototype payload; livestream video was achieved on the downcast, and the FOFL fiber optic broke upon retrieval. A similar result, with FOFL failure upon retrieval, was achieved using the same vessel in the upper pond of Narrow River (41.500168° N, −71.450029° W) on 22 July 2020.

For the first deepwater test, a 31′ center console monohull was used to access Atlantis Canyon (40.026958° N, −70.199236° W) on 19 August 2020. FOReels successfully transmitted live video throughout the cast to an estimated depth of 350 m (based on an acoustic depth sounder, no data logger was attached on this deployment) and was retrieved without breaking the FOFL fiber. Upon reaching the seafloor, live imagery of swarms of euphausiid shrimp, anemones, benthic fish, and other biology was viewed and recorded, including flashes of bioluminescence (Video S2). The FOReelS operator raised and lowered the payload based on visual feedback from the livestream output, similar to how a standard towed underwater vehicle can be operated. 

**Figure 4:**
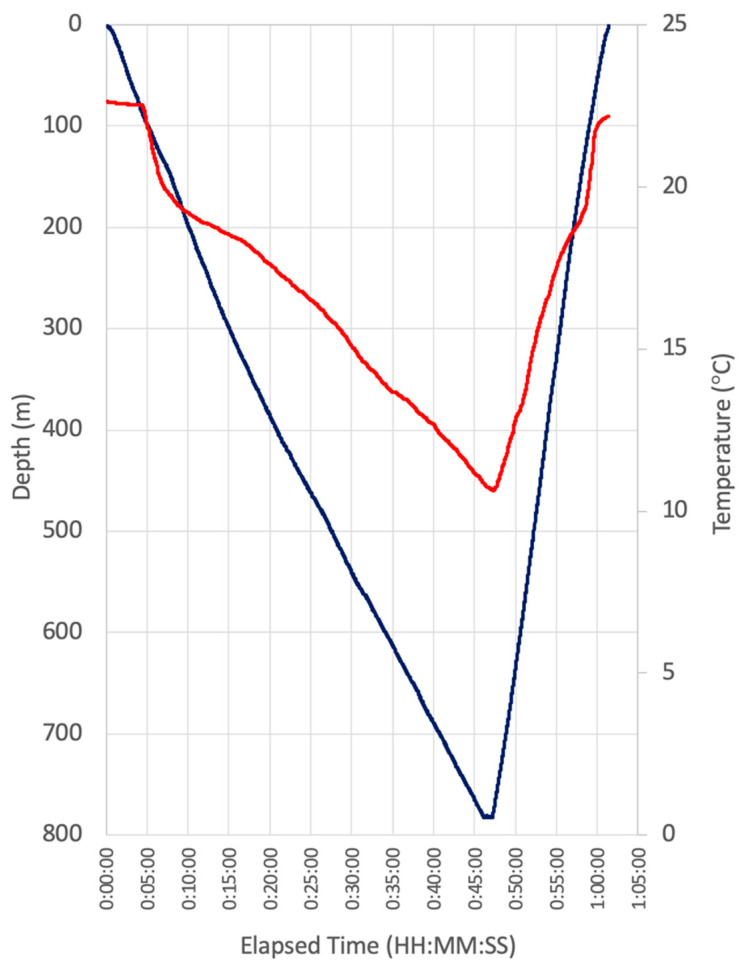
DPS depth and temperature profile from deepwater FOReelS deployment in Bermuda. See Supplementary Figure S1 for additional data of payload deployment behavior.

The second deepwater test of FOReelS took place on December 11, 2020 on the NE edge of Bermuda (32.354622° N, −64.547669° W) to a depth of 780 m using the 41′ R/V Henry Stommel. This cast took place in open deep water, and the payload was intentionally lowered to the fullest extent of the FOFL that was loaded on the reel (approximately 1500 m) while the vessel drifted along with the surface current at ~0.7 knots (0.36 m/s). A self-logging temperature/depth/3-axis tilt and compass sensor was attached to the payload and used to record the dive profile (Figure 4). Several interesting characteristics of the cast were observed using the data logger. By freespooling the payload for deployment (reel drag set to 0), it took a total of 46 min for the payload to reach the maximum depth of 780 m, corresponding with a consistent descent rate of 17 m/min. The upcast speed was much faster when powered by the reel, with an ascent rate of 41.8 m/min. The DPS spun in circles in mostly one direction throughout the descent, but maintained a consistent heading during recovery (Supplementary Figure S1). While little biology was observed during this cast, video was livestreamed throughout the deployment and the payload was again retrieved without breaking the FOFL.

## 4. Discussion

The FOReelS system is the result of a line of research and development into lightweight small form-factor deep-sea robots. The fishing reel instrument deployment concept was first developed for deep-sea exploration to depths >1500 m using self-recording cameras and standard hollow-core braided fishing line [14], and further refined through collaborative efforts [15]. The DPS is based on 3D-printed epoxy-filled mold designs for the DEEPi camera, first described by Phillips et al. [13]. Building on this, the methods described in this paper for the epoxy-potted fiber optic transmitter in the DPS is, to our knowledge, the first of its kind; no prior examples of epoxy-potted fiber optics exist in the literature. Similarly, the epoxy-potted/3D-printed mechanical termination for the FOFL is also a novel contribution. 

The closest commercially available analog to the FOReelS system is the Underway CTD (Teledyne, Inc., UCTD), which uses a reel-based apparatus and small-diameter braided Spectra line to deploy/retrieve a self-logging payload that records depth, temperature and salinity to >1000 m depth while a vessel transits at speeds from 1–13 knots. While robust and retrievable, the UCTD does not transmit information in real time. ‘Hybrid’ ROV systems using free-spooled bare optical fiber schemes [3,4,5,6,7,10,16,17,18] do allow for real-time data transmission, but do not allow the retrieval/redeployment capability offered by FOReelS. Repeated deployment/retrieval cycles is enabled by both the small-diameter load-bearing features of FOFL, combined with the novel mechanical termination design described in this paper.

Currently there are several types of ‘ruggedized’, ‘tactical’, and ‘reinforced’ subsea fiber optic cables commercially available that can be used as small-diameter tethers. These include Ruggedized Optical Cable (ROC, Cortland Cable Company), DuraTAC (Tactical Fiber Systems), STFOC (Linden Photonics), BRU-series cables (Solifos AG Fiber optic Systems), and custom variants used by the academic and defense sectors. These high-performance fiber optic designs are indeed suitable for subsea data transmission, but all employ some kind of rubber, urethane, or steel strength member (or combinations thereof) to protect the fiber optic and increase the strength rating. FOFL utilizes a single layer of braided small-diameter outer strength component that is braided directly over the fiber optic without any flexible coating or binding agent. This unique and simple design offers several important features. First, it allows for an extremely small diameter, flexible cable that is comparable with tight-buffered fiber optic (250 micron diameter). Second, it allows for a mechanical termination design that is easily assembled and that discretely separates the outer strength member from the fiber optic core. Third, the outer strength component is water-permeable, allowing for the fiber optic to come into direct contact with the surrounding medium. This final feature has notable applications for fiber optic distributed temperature and acoustic sensing (DAS/DTS), a state-of-the art method for observing temperature and strain along a fiber optic in high spatiotemporal resolution. In DAS/DTS, any coating or cladding on the actual fiber optic has the potential to attenuate its sensing capability; in some cases, this is an acceptable and necessary tradeoff [19] but in other applications it may be advantageous to increase sensitivity and optimize time response. FOFL offers the ability to both enable data transmission over long distances under tensile loading, while also serving as a DAS/DTS sensor with a high response time to changing conditions.

## 5. Conclusions

There are a number of design improvements and future capabilities that FOReelS will benefit from. The fishing reel sheave design, which is currently a repurposed carbon-fiber bike wheel, is challenging to operate with such a lightweight tether design and often overruns itself, causing the FOFL to jump off. A future design is envisioned without a rotating sheave altogether, that will instead rely on a solid smoothly curved edge to guide the FOFL as it is deployed and retrieved. Other multi-sheave tensioner designs may also be considered. Future DPS designs for FOReelS will be based on smaller microprocessors, such as Raspberry Pi Zero computer modules, which draw significantly less power than the Raspberry Pi 4 shown in this paper. Combined with a fiber optic media converter, we anticipate quadrupling the power endurance of the DPS. Better lighting for imaging applications is desired, and may be enabled by using multiple potted LED arrays arranged in a pattern similar to that used by MBARI’s i2MAP midwater-imaging AUV [20]. Similarly, integrated sensors such as depth, temperature, attitude and heading that broadcast data in real-time are also feasible, as well as optical modem-based wireless networking components. The descent and ascent behavior of the DPS, specifically the spinning behavior observed in the Bermuda field trial, may be passively addressed with a more hydrodynamic shape and a weight balanced and/or uniform fin design. Multicamera arrays are also currently in development and could be easily integrated to allow for complementary views of both midwater and seafloor environments. It is an obvious future direction to enable mobility on the DPS towards extremely compact deep-sea robotic platforms that involve human-in-the-loop live control and navigation schemes. Deployment platforms such as autonomous surface vehicles (ASVs) and unmanned aerial vehicles (UAVs) may also be enabled for deep-sea exploration by the compact FOReelS design. Ultimately, we envision FOReelS being used for a broad range of current and future applications, facilitated by sharing its core design elements with the academic, commercial, and defense communities.

## 6. Patents

The fiber optic fishing line (FOFL) and mechanical termination design have been filed under US Provisional Application 63/151,176 “Fiber Optic Reel System”.

## Supplementary Materials

The following are available online at https://www.mdpi.com/article/10.3390/s21072526/s1, Figure S1: Data payload system (DPS) attitude and compass direction during deepwater deployment in Bermuda, Video S1: Tensile testing of fiber optic fishing line and mechanical termination. Video S2: Deep-sea field testing.
