# Supplementary figures and images for: The Fiber Optic Reel System: A Compact Deployment Solution for Tethered Live-Telemetry Deep-Sea Robots and Sensors
Source: Sensors (Basel). 2021 Apr 4;21(7):2526. doi: 10.3390/s21072526 (PMC8038450; doi:10.3390/s21072526)

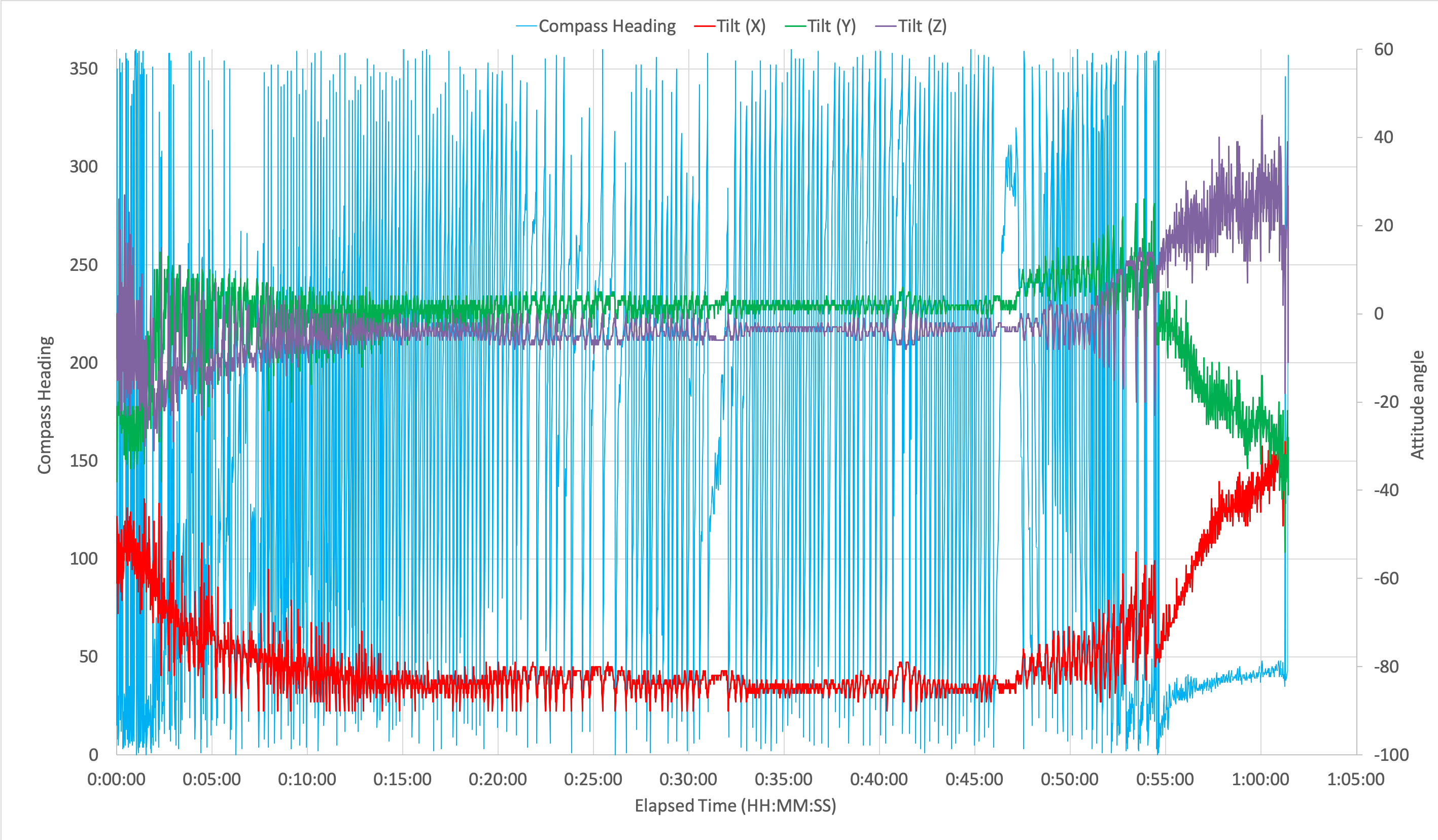

Supplement: Supplementary file 1 [file sensors-21-02526-s001.zip › Sup_Fig_1.png]
